# The Effects of *Melilotus officinalis* Extract on Expression of *Daxx, Nfkb and Vegf* Genes in the Streptozotocin-Induced Rat Model of Sporadic Alzheimer’s Disease

**Published:** 2017

**Authors:** Niloofar Bazazzadegan, Marzieh Dehghan Shasaltaneh, Kioomars Saliminejad, Koorosh Kamali, Mehdi Banan, Hamid Reza Khorram Khorshid

**Affiliations:** 1. Genetics Research Center, University of Social Welfare and Rehabilitation Sciences, Tehran, Iran; 2. Laboratory of Neuro-organic Chemistry, Institute of Biochemistry and Biophysics (IBB), University of Tehran, Tehran, Iran; 3. Reproductive Biotechnology Research Center, Avicenna Research Institute, ACECR, Tehran, Iran

**Keywords:** Alzheimer disease, Gene expression, Herbal medicine

## Abstract

**Background:**

Possible mechanisms of Alzheimer Disease (AD) such as inflammation and oxidative stresses in the brain led us to investigate potential AD therapeutics of *Melilotus officinalis,* an herbal extract, with possible role as an anti-inflammatory and anti-oxidant agent. Among different genes which had important role in Sporadic AD (SAD), three genes including *DAXX, NFkB* and *VEGF* have shown significant statistical diversity in the brains of Alzheimer patients.

**Methods:**

These genes were chosen to be investigated for neuroprotective effects of the extract by comparing the expression level in the hippocampus of Sporadic AD (SAD) rat model using quantitative polymerase chain reaction (qPCR) in the treated and untreated groups. In addition, therapeutic effects at the behavioral, learning and memory level by Morris Water Maze (MWM) test were investigated.

**Results:**

The results represented significant decreased expression in *Daxx, Nfkb* and *Vegf* genes in the SAD rat’s model treated with the herbal extract compared to the Streptozotocin-induced (STZ-induced) rats. Furthermore, no significant changes were seen in swimming distance and time for finding the hidden platform in the herbal-treated compared to the STZ-induced group. In memory level, no significant changes were observed among treated and untreated groups.

**Conclusion:**

It seems that the herbal extract may have significant effect on Alzheimer-related gene expression changes but not on clinical levels.

## Introduction

Alzheimer Disease (AD) is the most common form of dementia with a predicted global incidence of 1 in every 85 cases by 2050 ^1^. It is characterized by progressive memory impairment and diminished cognitive performance which finally leads to death ^2,3^. The major pathological findings of AD brain are senile plaques, largely composed of extracellular amyloid-β (Aβ) peptides, and Neurofibrillary Tangles (NFTs), consisting of polymerized hyperphosphorylated tau protein. Sporadic or late-onset AD (>65 years) is the most common form of this disease, accounting for over 95% of cases ^4,5^.

Finding an effective method to prevent or slow down the disease process is a significant clinical challenge in treatment of Alzheimer’s disease. Cholinesterase inhibitors have a limited clinical effect on the Alzheimer’s symptoms ^6^. For years, drug developers focused on acetylcholinesterase inhibitors, with tacrine as the class leader ^7^. It showed relatively moderate activity in clinical trials; however, its therapeutic use was interrupted because of liver toxicity, which required close monitoring of patient’s liver function ^7^. A new generation of acetylcholinesterase inhibitors, galantamine, donepezil, and rivastigmine with no liver toxicity were released in the market; however, therapeutic advantages were questionable ^8^. Animal models of AD play important role in the process of drug development. Their function mimics the disease in humans. Based on the hypothesis that is demonstrative of AD as type 3 diabetes which showed a consistent decrease in expression of insulin, insulin-like growth factors and their receptors in brains of AD patients, injection of STZ into the rat brain results in the same pattern ^9^. STZ causes phosphorylation of the tau protein, amyloid deposits, cognitive impairment, insulin desensitization and neuronal death ^10,11^. This AD rat model provides sporadic form of AD and would be appropriate in drug research. Herbal medicine in treatment of dementia in China represented some cognitive benefits ^12^.

Other studies on AD therapy by herbal medicine showed that active ingredient effects of these herbs on disease are not limited to the inhibition of cholinesterase inhibitors and include the modification of Aβ processing, protection against apoptosis and oxidative stress and anti-inflammatory effects ^13^.

*Melilotus officinalis* or yellow sweet clover is a species of a plant in the family of *Feabaceae*. It contains coumarin, flavonoides (kampeferol, quercetin glycosides), triterpene saponins and volatile oil with anti-inflammatory, antioxidant and neuroprotective effects ^14–16^. Moreover, this herbal extract can improve diabetic foot ulcers, and its usefulness in microcirculation improvement has been reported ^17,18^. This extract decreases nitric oxide synthesis which has important effects on bone cell function ^15,19^.

Among many genes related to the pathology of SAD, *DAXX*, *NFκβ* and *VEGF* genes with the role in apoptosis, inflammation and angiogenesis showed significant statistical diversity in human brain with Alzheimer ^20^. Regarding AD, possible mechanisms such as inflammation and oxidative stresses in the brain, and the role of different genes in these processes, the neuroprotective effect of this herbal extract was studied by comparing the expression levels of these genes in the hippocampus of rat model of Sporadic AD (SAD) using qPCR in treated and untreated groups. Additionally, the therapeutic effects were investigated at behavioral, learning and memory level as well.

## Materials and Methods

Thirty six adult male *Wistar* rats with weight of 250–300 *g* were used in this research. They were maintained in cage with enough food and water, in a constant environment at 22*°C* and 12 *hr* light/dark cycle according to the previous study ^21^. Animals were divided into five groups each consisting of six to eight rats: the control group (eight rats) without any medication and surgery; the sham group (eight rats) which received bilateral intracerebroventricular (ICV) injection of aCSF as the vehicle of STZ; the Alzheimer group (seven rats) with bilateral ICV injection of STZ (3 *mg/kg*); the ethanol-treated STZ group (six rats), which received diluted ethanol 86% (10 fold dilution) as I.P. as the vehicle of herbal extract ^21^ and the herbal extract treated STZ group (seven rats) which received the component as intraperitoneal injection at the dose of 20 *mg/kg/day* for 21 days after modeling ^21^.

All groups of rats were tested for learning and memory using Morris Water Maze (MWM) test ^22^. Then, they were sacrificed with stereotaxic surgery and all hippocampi were dissected and stored in RNA protector solution at −20*°C* ^23^. All procedures were performed according to the National Institute of Health Guide for the care and use of laboratory animals ^24^.

Total RNAs were extracted from hippocampus tissues using UP100H ultrasonic processor (Germany) and RNeasy Plus Mini Kit (Qiagen, Hilden, Germany) according to the manufacturer’s protocol. Purity and integrity of RNAs were assessed using Nano-drop spectrophotometer and RNase-free gel electrophoresis. cDNA synthesis was performed using RevertAid^TM^ First Strand cDNA Synthesis Kit (Fermentas, Thermo Fisher Scientific) according to the manufacturer’s protocol.

SYBR green Real Time PCR was used to detect the relative expression levels of the three genes *Daxx, Nfkb* and *Vegf* in rat hippocampus of each group. ABI 7500 Real-time PCR system (Applied Biosystem, Foster city, CA, USA) with Takara SYBR Master Mix (Shiga, Japan) was used in our study. All gene expression normalizations were performed by *Actb* endogenous control ^25,26^. Cycle threshold (Ct) values were used to calculate fold changes in gene expression between groups using REST 2009 software. P-values less than 0.01 for analysis by REST and in other analysis less than 0.05 were considered statistically significant. MWM test data were analyzed by GraphPad Prism 6 software; Kruskal Wallis (Dunn’s multiple comparisons test) test was used for three recorded factors (path length, escape latency and swimming speed) in all treated and untreated groups separately during five days.

## Results

### Behavioral test

**Figure 1.**
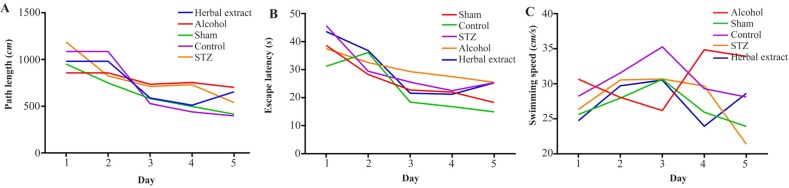
The mean value of path length (swimming distance), escape latency (time for finding hidden platform) and swimming speed (velocity) during five continuous days in all treated and control groups represented in 1A, 1B and 1C, respectively.

**Figure 2.**
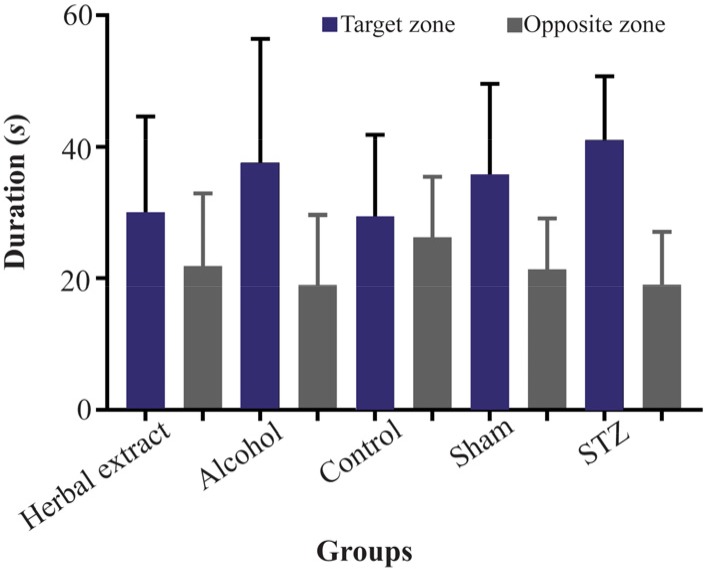
Probe test assesses inability of rats to find the hidden platform. The median with interquartile range of percentage of time spending in target and opposite zone on the 6th day (four trials) of the test in all treated and control groups.

After evaluating the learning and memory level changes during the trial days by Morris Water Maze test, our results showed that there was a significant decrease in swimming distance and time for finding the hidden platform in each group during five days except in alcohol group. Figures 1A, B and C are representative of MWM test analyses among treated and untreated groups (Figure 1). The swimming speed did not show any significant change during five trial days among groups. Probe test results showed no significant memory increasing in the herbal-treated compared with the STZ-induced group but showed the same level as did the control group (Figure 2).

### Gene expression

**Figure 3.**
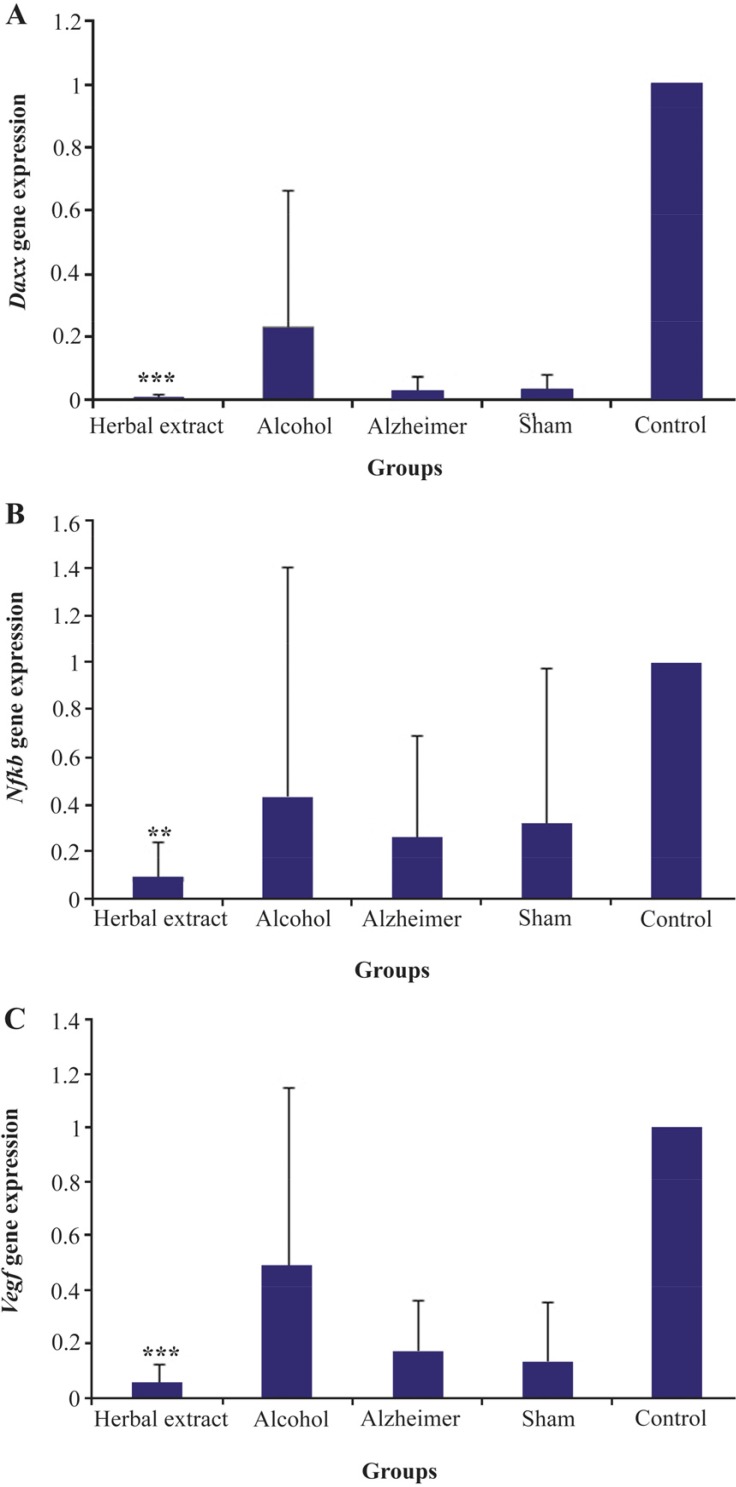
Expression level of *Daxx, Nfkb* and *Vegf* in treated and control groups has been presented in figures 3A, 3B and 3C, respectively. Significant expression changes of herbal-treated compared to Alzheimer group were labeled out with asterisk.

*Daxx*, *Nfkb* and *Vegf* genes were down-regulated in rat’s hippocampus after three weeks of treatment with herbal extracts comparing to the Alzheimer group without treatment. Statistically significant change (p≤0.003) was observed in expression level of *Daxx*, *Nfkb* and *Vegf* with *a* decrease of about three-fold (Figure 3). The expression of both *Nfkb* and *Vegf* significantly decreased in the model compared to the control group with p-values of 0.008 and 0.000, respectively.

## Discussion

In this study, the expression of three candidate genes was evaluated (*Daxx, Nfkb* and *Vegf*) for Alzheimer’s disease in RNA level in rat models of AD. Our results showed that all three genes, *Daxx*, *Nfkb* and *Vegf* were significantly down-regulated in the herbal extract compared to the model group. DAXX protein was related to FAS protein which belongs to the tumor necrosis factor receptor superfamily and its intracellular tail contains death domain which is critical for apoptotic signaling. DAXX binds to the death domain and over-expression of DAXX increases apoptosis ^20^. According to the previous study, these genes were up-regulated in AD brain ^20^. Despite the reports supporting a pro-cell death function for Daxx, many studies have proposed a potential anti-apoptotic function for it ^27^. As it is clear in figure 3A, *Daxx* in the STZ-induced compared to the control group was down-regulated. One possible explanation for this finding could be due to the early stages of the disease which can have effect on expression level of gene.

NF-κB is a pro-inflammatory transcription factor which is increased in AD brain. It is oxygen sensitive and also is a precursor to *VEGF* gene expression that leads to angiogenesis. Activation of NF-κB can protect neurons against death inferred by amyloid β-peptide ^28^. NF-κB might also play a role in amyloidogenesis itself, since expression of APP can be due to NF-κB ^29^. The ICV-STZ model presents many features of abnormalities seen in SAD brain, but does not develop Aβ plaques or NFTs ^30^. There is the possibility that *VEGF* might be activated in AD ^31^. *VEGF* levels in AD patient have been controversial. Increased levels of this gene in the hippocampal cortex of AD patients compared with normal brain were reported ^32^. According to the available data, it is postulated that reduced *VEGF* expression might be proved in AD ^33^. Other genetic studies have defined that *VEGF* levels cause neurode-generation in part by damaging neural tissue perfusion ^34^. Gene array analysis has shown upregulation of angiogenesis relevant genes in the AD brain ^35^. In the present study, the expression of both *Nfkb* and *Vegf* significantly decreased in the model compared to the control group with p-values of 0.008 and 0.000, respectively. It might reflect the effect of AD stage on expression level of genes. Similarly, in the herbal-treated compared to model group, all three genes, *Daxx*, *Nfkb* and *Vegf* showed significant down-regulation (p-value =0.000, 0.003 and 0.000). *Vegf* expression is dependent on two other genes expression. Altogether, *Daxx* and *Nfkb* down-regulation in the herbal extract-treated group could be indicative of the extract effect on apoptosis and inflammation, respectively. Also, *Vegf* gene down-regulation in the herbal-treated group may be effective in decreasing angiogenesis. Regarding MWM results, there was a significant decrease in the swimming distance and time for finding the hidden platform in each group during five days except alcohol group. Furthermore, no significant memory change was observed among treated and untreated groups, but memory level of herbal-treated was the same as control group.

## Conclusion

In conclusion, regarding both behavioral and gene expression results, it could be concluded that this herbal extract may have significant effects on gene expression level.
